# Experimental Research on Gradation Range and Performance of SMAC13

**DOI:** 10.3390/ma17112680

**Published:** 2024-06-02

**Authors:** Qianqian Zhen, Weidong Cao, Rui Dong, Shutang Liu, Ning Liu, Zunhao Zhan, Yingjian Li

**Affiliations:** 1 Shandong Hi-Speed Infrastructure Construction Co., Ltd., Jinan 250001, China; 13808928496@163.com (Q.Z.); dongrui1986@163.com (R.D.); maiweimila7@163.com (N.L.); 2 School of Qilu Transportation, Shandong University, Jinan 250002, China; 202335518@mail.sdu.edu.cn (Z.Z.); 202335505@mail.sdu.edu.cn (Y.L.)

**Keywords:** SMAC 13, gradation range, performance, stone matrix asphalt (SMA), asphalt concrete mixture (AC)

## Abstract

Stone matrix asphalt and asphalt concrete mixture with 13.2 mm nominal maximum aggregate size (named SMA13 and AC13, respectively) are widely used in the surface course of asphalt pavement in China. Generally, the pavement performance of SMA13 is superior to that of AC13, while the cost of the former is significantly higher than that of the latter. The objective of this paper was to develop a new hot mix asphalt (named SMAC13) whose performance and cost are between SMA13 and AC13. A boundary sieve size (BSS) of 2.36 mm was selected between fine and coarse aggregates. Based on the union set of aggregate gradation ranges of SMA13 and AC13, the family of gradation curves in the forms of *S* shapes were designed in terms of the BSS passing rate. According to the evaluation of the skeleton interlock of coarse aggregate of the gradation curve family, the aggregate gradation range of SMAC13 was determined. Also, the performance of three kinds of asphalt mixtures were compared through laboratory tests. The results indicated that SMA13 shows the best rutting resistance, followed by SMAC13 then AC13, while in terms of low-temperature performance in resistance to cracking, the sequence is SMAC13, AC13, and SMA13. The sequence of water stability is AC13, SMAC13, and SMA13. Furthermore, the cost of SMAC13 is 25% less than that of SMA13. Therefore, SMAC13 can be used as an alternative for the surface course of asphalt pavement in terms of performance and cost.

## 1. Introduction

Asphalt mixes are classified into three structural types—skeleton-void structures, skeleton-dense structures, and suspension-dense structures—based on mineral gradation composition and air voids. Typically, skeleton-dense asphalt mixes exhibit superior mechanical strength and high-temperature stability [1,2]. Therefore, designing the gradation of a skeleton-dense asphalt mix has emerged as a significant topic of interest and a critical research issue [3,4,5]. Stone matrix asphalt (SMA) and asphalt concrete mixture (AC) are the two most commonly used types of mixes in highways and urban road pavements in China and other countries. It is well known that SMA is a typical skeleton-dense asphalt mix, and it has been widely used for the top layer of asphalt pavements in many countries around the world due to its excellent high-temperature rutting resistance, low-temperature cracking resistance, water damage resistance, fatigue resistance, and skidding resistance [6,7]. Traditionally, AC is considered a suspension-dense asphalt mixture with good water stability and durability. However, it often lacks sufficient high-temperature rutting resistance and skidding resistance. The aggregates used for AC are generally low-cost limestone and other stone materials that are widely distributed in many regions. Also, the amount of asphalt binder used is relatively small, and unmodified asphalt without the addition of fibers is usually adopted. Consequently, the cost of AC is relatively low. AC can be used in base, intermediate layers and the surface of a pavement structure [8].

Compared to AC, SMA typically contains a higher content of coarse aggregate, mineral filler, and asphalt binder, as well as less fine aggregate. Additionally, fiber stabilizers need to be added [2], leading to a significantly higher cost. To ensure the higher rutting and skidding resistance of SMA, basalt and other high-quality stones are often used as coarse aggregate. However, with the enlargement in the scale of expressway construction in China in recent years, the rising demand for basalt stone has led to soaring costs in SMA production. Some researchers investigated the utilization of hybrid lithological stone or steel slag in SMA to mitigate the cost by reducing the usage of basalt coarse aggregate [9,10,11,12,13,14,15,16,17,18]. However, the long-term performance of these SMAs needs to be verified through engineering practice. Reducing the asphalt content is a possible way to further decrease the cost of asphalt mixes. To achieve this reduction, it is necessary to adjust the aggregate gradation. A semi-dense asphalt (SDA) mixture with a porosity of 12–16% is primarily used in Switzerland for its low-noise properties [19]. Although SDA mixes had been designed and applied in China and other countries, they have hardly ever been used in recent decades due to concerns about premature distress and insufficient durability in China. Therefore, dense-graded and gap-graded asphalt mixes are still dominant mixtures used in pavement structure in China and many other countries. There are few reports on dense gap-graded asphalt mix, the performance and cost of which are between SMA and AC. Comprehensively considering performance and cost, a new asphalt mixture called SMAC13 was developed in this study. The research focused on asphalt mixes with a nominal maximum particle size of 13.2 mm, commonly used in surface layers of asphalt pavement. The mineral aggregate gradation range of SMAC13 was between those of SMA13 and AC13, with the aim of matching the former’s performance as closely as possible. The paper can provide a new scheme for the selection of asphalt pavement surface for high-grade highways and urban roads.

## 2. Raw Materials and Basic Properties

### 2.1. Aggregates

Two kinds of coarse aggregates, consisting of limestone and basalt, were used in the laboratory. Each kind of coarse aggregate included two particle sizes, i.e., 5–10 mm and 10–15 mm. Basic properties of coarse aggregates were tested as per Chinese standards [20], and the results are shown in Table 1.

**Figure 1:**
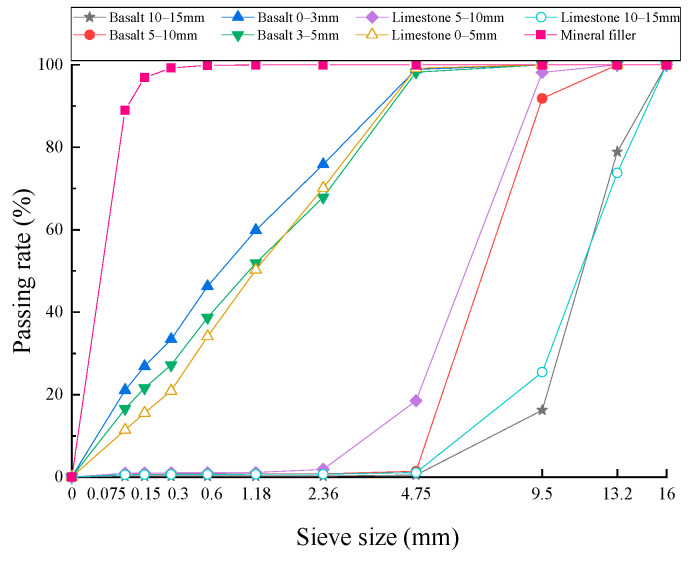
Sieving curves for aggregates and mineral filler.

The fine aggregates also consisted of limestone and basalt. The fine basalt aggregate was further classified into two kinds of particle sizes, 0–3 mm and 3–5 mm, while the particle sizes of the fine limestone aggregate ranged from 0 to 5 mm. The basic properties of the fine aggregates are shown in Table 2. The mineral filler used was limestone powder, and its basic properties are shown in Table 3. The results of the sieving test for each aggregate and mineral filler are presented in Figure 1. All aggregates and mineral fillers met the technical requirements of Chinese specifications [21].

### 2.2. Asphalt and Fiber

SBS-modified asphalt from a petrochemical company in Shandong Province was used. The basic properties of this SBS-modified asphalt are shown in Table 4. The results met specifications for modified asphalt binders [21].

The fiber selected for the test was lignin, which acted as a stabilizer for asphalt binder and reinforcement in the asphalt mix. Its basic performance indicators on testing are shown in Table 5, and met the relevant technical requirements of the specification [21].

## 3. Family of Gradation Curves and Gradation Range

### 3.1. Determination of the Boundary Sieve Size of Aggregates

The boundary sieve size (BSS) between fine and coarse aggregates of SMA and AC are determined by the nominal maximum particle size (NMPS) of mixture in China specification [21]. If the NMPS for SMA is less than or equal to 9.5 mm, then the BSS is 2.36 mm. If the NMPS is greater than or equal to 13.2 mm, the BSS for SMA is 4.75 mm. For AC, if the NMPS is less than or equal to 16 mm, the BSS is 2.36 mm. However, if the NMPS is greater than or equal to 19 mm, the BSS is 4.75 mm. Based on previous research [22,23], the fine material, which is composed of fine aggregate and filler, is used to fill the gaps in the coarse material skeleton. To minimize the interference of fine aggregate with the interlocking of coarse aggregate, the BSS should not be larger. Considering that NMPS of the asphalt mixtures used in this study is 13.2 mm, the BSS selected was 2.36 mm. Moreover, if the aggregate particles greater than or equal to 2.36 mm are considered the skeleton of coarse aggregates, AC can also be designed as a skeleton-dense gradation [24].

### 3.2. Design of Family of Gradation Curves

**Figure 2:**
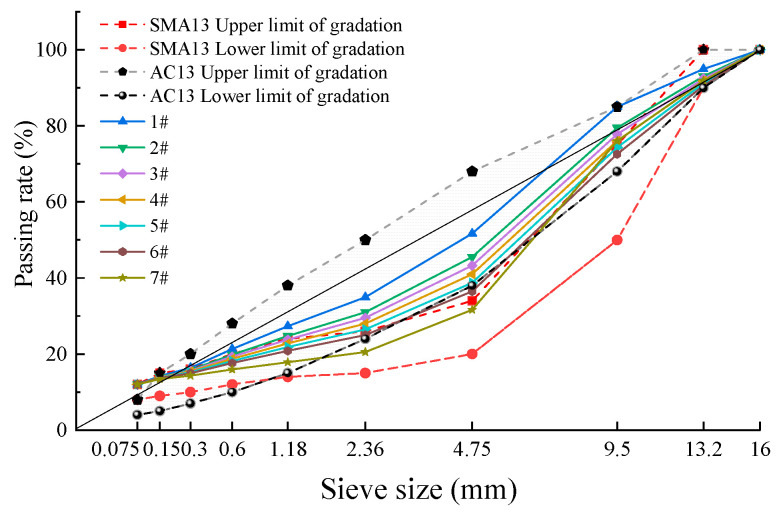
Proposed family of gradation curves for SMAC13.

To investigative the gradation range of SMAC13, seven gradation curves were tentatively designed in the union set of the upper and lower gradation ranges of SMA13 and AC13 in the specifications [21], which constituted the family grading curves of SMAC13. In theory, a skeleton-dense structure of a mixture can be formed by mineral aggregates when the proportion of coarse and fine aggregates is suitable. Furthermore, an S-shaped curve is preferable for the gradation curves [23,25]. Thus, the family of gradation curves were designed by adjusting the passing rate of BSS in S-shaped curves. Seven gradation curves were numbered gradations 1–7 according to the passing rate of BSS, from the largest to the smallest, as shown in Table 6 and Figure 2.

### 3.3. Determination of Asphalt-Aggregate Ratio and Volume Index

After the family of gradation curves of SMAC13 are determined, the initial asphalt-aggregate ratio can be estimated using Equation (1) [26]:

(1) $$P_{a}=\left(\frac{100-VV}{100-VMA}\times \frac{1}{\gamma_{\mathrm{sb}}}-\frac{1}{\gamma_{\mathrm{se}}}\right)\times \gamma_{a}\times 100$$

where $P_{a}$ is the initial asphalt aggregate ratio (%), *VV* the air voids (%), *VMA* the voids in mineral aggregate (%), $\gamma_{se}$ the effective specific gravity of mineral aggregate, $\gamma_{sb}$ the bulk specific gravity of mineral aggregate, and $\gamma_{a}$ the specific gravity of asphalt binder, and the test value is 1.02.

In Equation (1), $\gamma_{sb}$ and $\gamma_{se}$ can be calculated according to the proportion of each gradation and density parameter of raw materials. Based on the requirements for VV and VMA of SMA13 and AC13 in the specifications [21], VV is set at 4.0% and VMA is set at three values, i.e., 14%, 14.5%, and 15%. The calculation results of $P_{a}$ are shown in Table 7.

Since the asphalt aggregate ratio calculated by Equation (1) is a theoretical estimated value, it needs to be verified. Gradation 2 was selected as an example, and specimens with asphalt aggregate ratio of 5.3% were prepared using the Marshall process. The measured average values of VV and VMA of the specimens were 2.4% and 12.8%, respectively, which were smaller than the set values. Therefore, the estimated asphalt aggregate ratio may be larger than the desired value (i.e., optimum asphalt aggregate ratio). The asphalt aggregate ratio can be adjusted by referring to Equation (2) in Superpave [27]:

(2) $$P_{oe}=P_{a}-0.4\times \left(4-VV\right)$$

where $P_{oe}$ is the estimated asphalt aggregate ratio at VV of 4% (%), $P_{a}$ the asphalt aggregate ratio used in the test (%), and VV the measured air voids of Marshall specimens (%).

**Figure 3:**
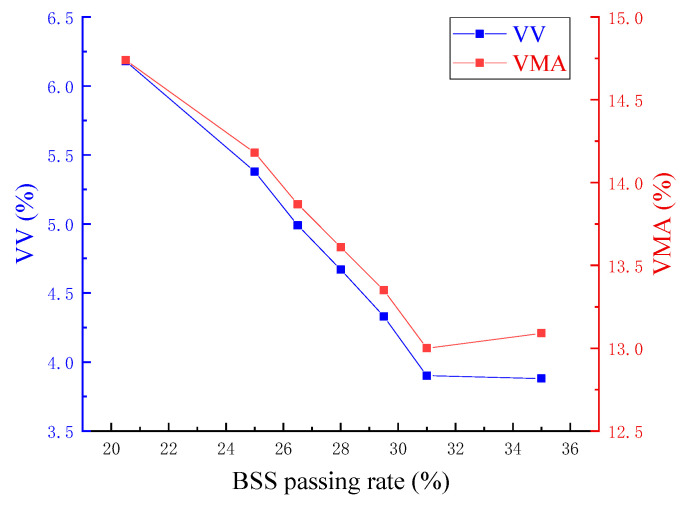
Effect of the BSS passing rate on VV and VMA.

The estimated asphalt aggregate ratio at VV of 4% for gradation 2 was 4.7% by calculation with Equation (2). Then, Marshall specimens of gradation 2 were re-prepared with 4.7% asphalt aggregate ratio. The test results of VV and VMA were 3.9% and 13%, respectively, which were close to the target values. To reduce experimental workload and compare the volumetric indexes of gradation curve family under the same asphalt content, an asphalt aggregate ratio of 4.7% was also employed for the remaining gradations. The variation rules of key volumetric indexes of VV and VMA for various passing rates of BSS corresponding to family of gradation curves are shown in Figure 3.

From Figure 3, it can be seen that the ranges of VV and VMA are 3.5–6.2% and 13.0–15.0%, respectively, which can satisfy the requirements of specifications for AC13 by and large. The trends of VV and VMA for passing rates of BSS are almost the same, which initially decrease and then increase with the increase in BSS passing rates. It is evident that the VV and VMA values approach the valley points when the passing rate reaches about 31%. This indicates that an ideal skeleton-dense structure of asphalt mix is achieved at this passing rate. As the passing rate decreases, the degree of density on the left side of the valley point diminishes and shifts towards a skeleton-gap structure. Conversely, as the passing rate increases, the mixture on the right side of the valley point transforms into a suspension-dense structure.

### 3.4. Determination of the Boundary Sieve Size of Aggregates

#### 3.4.1. Determination of Skeleton Interlock

The interlock properties of SMAC13 can be assessed according to the standard for determining the extent of interlock in SMA. If the value of voids in the coarse aggregate of the asphalt mix (VCA_mix_) is less than that of the voids in the coarse aggregate (VCA), the interlocking skeleton (i.e., stone-on-stone skeleton) can be achieved. VCA_mix_ was calculated for each gradation using Equation (3). The VCA can be measured through vibrating compaction testing of coarse aggregate using a vibration table [28]. The results of the calculated VCA_mix_ and values of VCA are shown in Table 8.

(3) $$VCA_{\mathrm{mix}}=\left(1-\frac{\gamma_{f}}{\gamma_{\mathrm{ca}}}\times \frac{P_{\mathrm{CA}}}{100}\right)\times 100$$

where ${VCA}_{mix}$ is percentage void in coarse aggregate of asphalt mix (%), $\gamma_{f}$ is the bulk specific gravity of compacted asphalt mix, $P_{CA}$ is the mass percentage of coarse aggregate by asphalt mix, and $\gamma_{ca}$ is the synthetic bulk specific gravity of coarse aggregate.

In Table 8, it can be seen that the VCA_mix_ values of gradations 2–7 are less than VCA values, indicating that these gradations can form an interlocking skeleton structure.

#### 3.4.2. Gradation Range Determination

**Figure 4:**
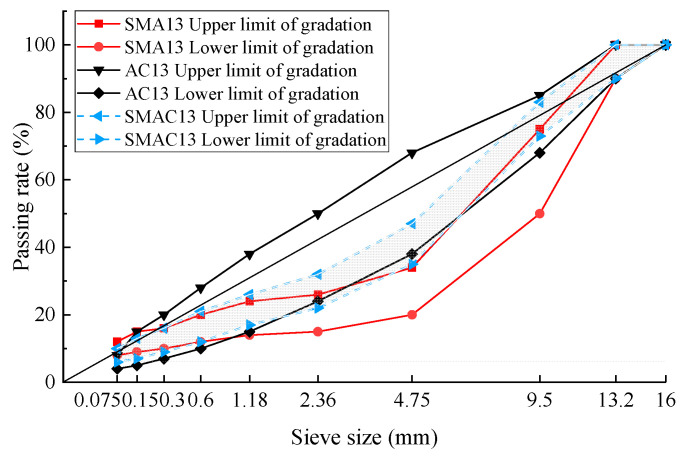
Comparison of gradation ranges for SMAC13, AC13, and SMA 13.

According to the definition of SMAC13 in this study, the basic requirement of gradation is that the interlocking skeleton should be formed within the gradation range as much as possible. Therefore, based on the BSS passing rates of gradations 2–7, the range of BSS passing rates of SMAC13 was determined to be 22% to 32% (the upper limit is slightly wider than the passing rate for gradation 2). The range of passing rates of NMPS 13.2 mm was determined to be 90% to 100% based on SMA13 gradation. The range of the 0.075 mm passing rate was 6% to 10%, which was determined by averaging the upper and lower gradation limits of AC13 and SMA13 with a 0.075 mm sieve. Other passing rates for the remaining sieve sizes were recommended in terms of S-shaped gradation distribution. The finalized SMAC13 gradation ranges are shown in Table 9. A visual comparison of the gradation ranges of SMAC13, AC13, and SMA13 is shown in Figure 4.

In Figure 4, it can be seen that the gradation patterns of SMAC13 show obvious S-shaped curves that are similar to those of SMA13. On the whole, the middle-sieve-size section (2.36 mm–9.5 mm) of the gradation range for SMAC13 is above the upper limit of gradation for SMA13, while the tail section (2.36 mm–0.075 mm) and head section (9.5 mm–16 mm) are among the gradation range of AC13.

## 4. Performance Evaluation of SMAC13

### 4.1. Mix Design

**Figure 5:**
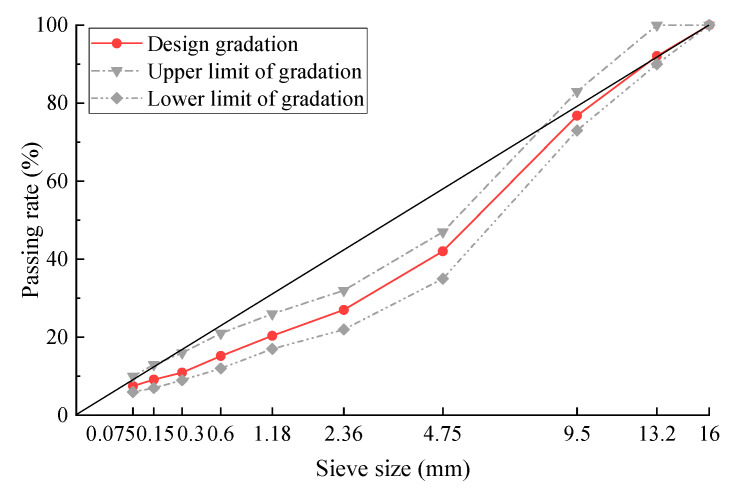
Gradation curve of SMAC13.

To ensure the representativeness and comparability of the designed gradations of the three kinds of asphalt mixes (SMAC13, AC13, and SMA13), the proposed gradation curves are positioned in or close to the middle of their respective gradation ranges. The design gradation curve for the SMAC13 is illustrated in Figure 5.

The asphalt aggregate ratio was estimated using Equations (1) and (2), and the result was 5.8%. Then, Marshall specimens were prepared with the determined asphalt aggregate ratio and volumetric parameters were tested to study the effect of the addition of fiber on volume indexes and drain-down. The results of the tests are presented in Table 10. In Table 10, fiber content is the percentage of the mass of fiber by the total mass of mineral aggregate, $\gamma_{t}$ is the maximum theoretical specific gravity of specimens, and $\gamma_{b}$ is the bulk specific gravity of specimens.

As shown in Table 10, the drain-down of the asphalt mixture without the addition of fiber exceeds the technical requirements of the specification for SMA, while the addition of 0.2% fiber can obviously reduce drain-down, as well as reduce the VV to some extent. Therefore, the addition of 0.2% fiber for SMAC is suggested. Since VV is 3.58% at an asphalt aggregate ratio of 5.8%, the optimum asphalt aggregate ratio at VV of 4% can be determined through Marshall tests according to the specifications [21]. The optimum asphalt aggregate ratio at VV of 4% was determined to be 5.5%.

**Figure 6:**
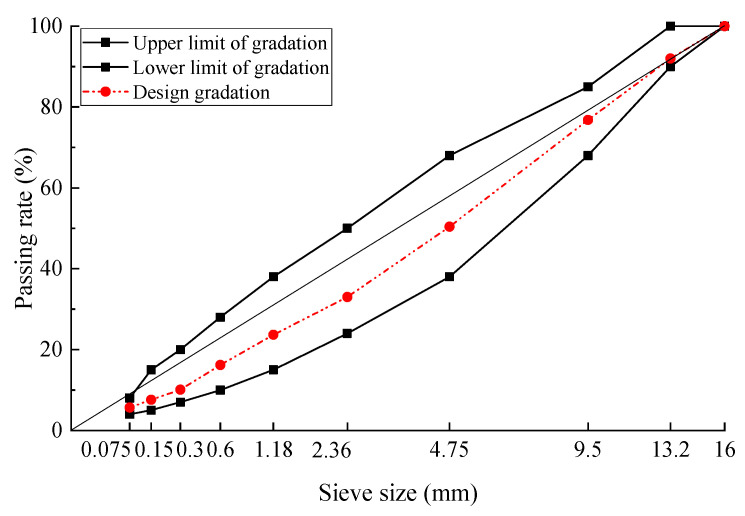
Gradation curve of AC13.

The gradation of AC13 was designed using limestone aggregate, and the gradation curve is shown in Figure 6. The optimum asphalt aggregate ratio was determined to be 5.0% according to the design method for AC in the specifications [21].

**Figure 7:**
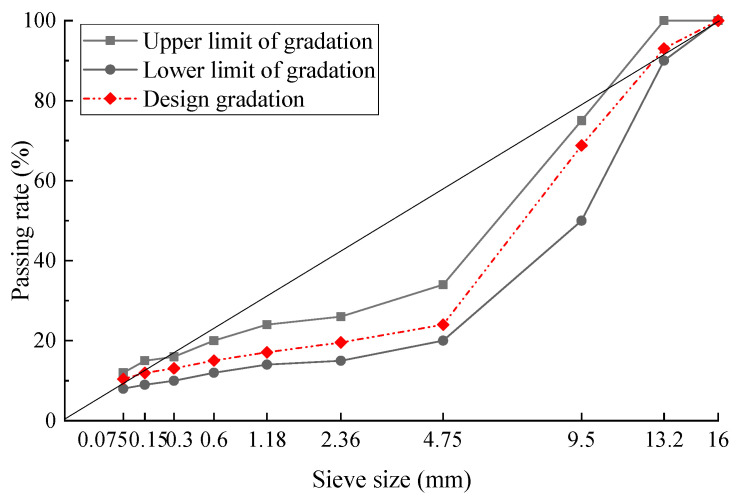
Gradation curve of SMA13.

The gradation of SMA13 was designed using basalt aggregate and 0.3% lignin fiber, and the design gradation curve was shown in Figure 7. The optimum asphalt aggregate ratio was determined to be 6.2% according to the design method for SMA in the specifications [21].

### 4.2. Performance Comparison and Analysis

#### 4.2.1. High-Temperature Performance

**Figure 8:**
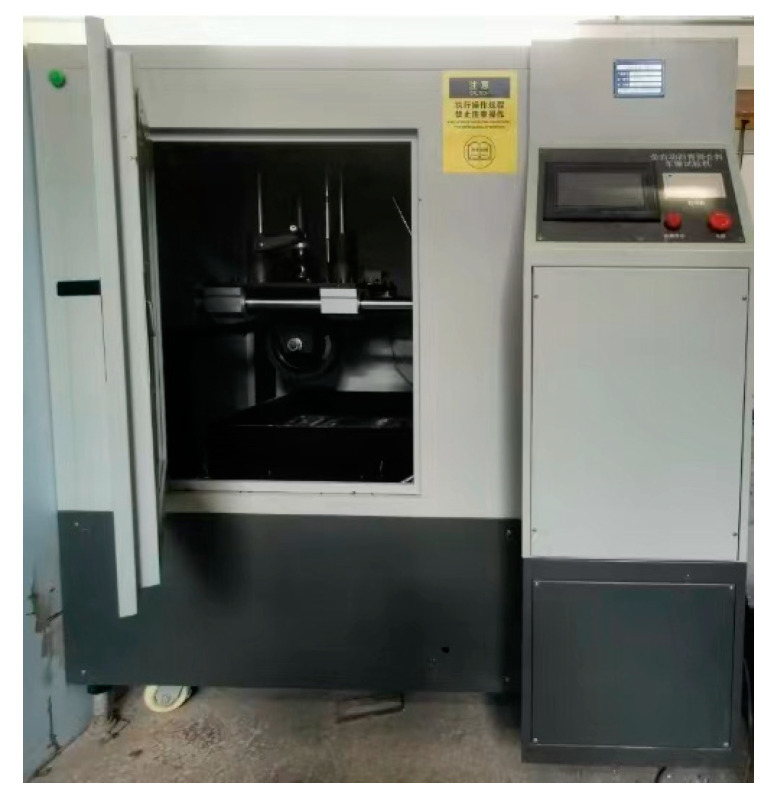
Wheel tracking tester.

**Figure 9:**
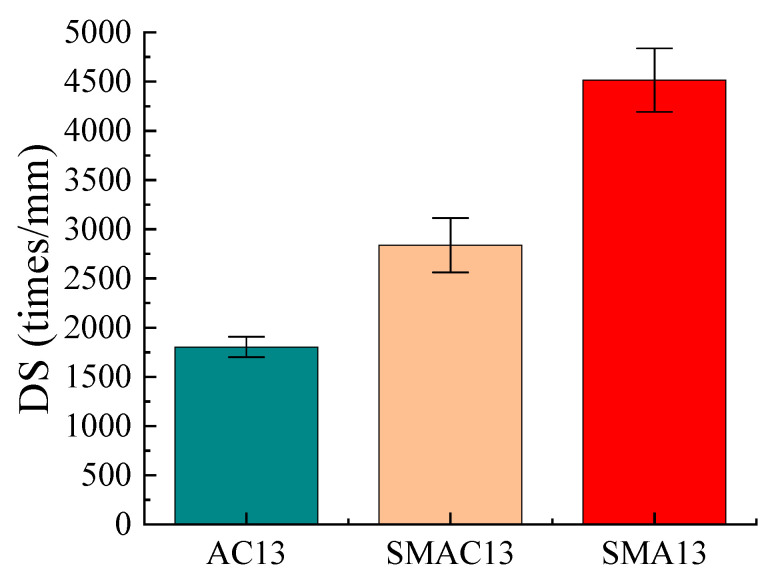
Results of wheel tracking test.

**Figure 10:**
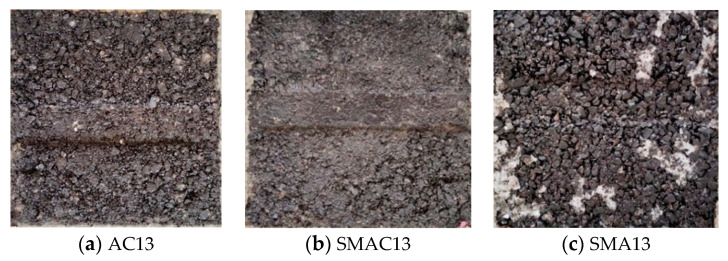
Specimens of three types of asphalt mix after testing.

The wheel tracking test was used to evaluate the high-temperature rutting resistance of asphalt mixes. Laboratory equipment used was shown in Figure 8, and the test was conducted as per China standards [29]. The test index was dynamic stability (DS), and the test temperature was 60 °C. Three test specimens were used for each kind of asphalt mix. Figure 9 displays the wheel tracking test results for the three different types of asphalt mixes. Based on dynamic stability values, the three types of asphalt mixes’ high-temperature performance were ranked SMA13, SMAC13, and AC13. The dynamic stability of SMAC13 is 2839 times/mm, which meets the requirements for modified asphalt mixtures in the specifications [21]. The values of dynamic stability for SMAC13 and SMA13 are 1.57 times and 2.50 times that of AC13, respectively. Therefore, SMAC13 has better rutting resistance than AC13. This can be attributed to the skeleton-dense structure and the addition of fiber in SMAC13. On the other hand, because of the basalt aggregates and 0.3% fiber in SMA13, the rutting resistance of SMAC13 is lower than that of SMA13. The specimens for each kind of asphalt mix after testing are shown in Figure 10.

#### 4.2.2. Low-Temperature Performance

**Figure 11:**
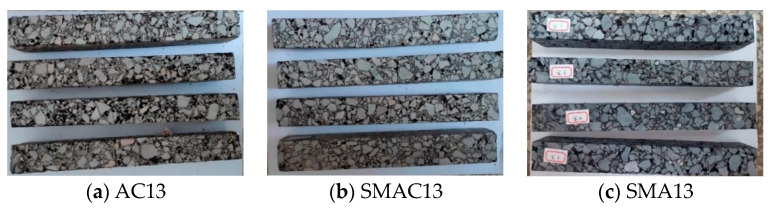
Test specimens of three types of asphalt mix.

**Figure 12:**
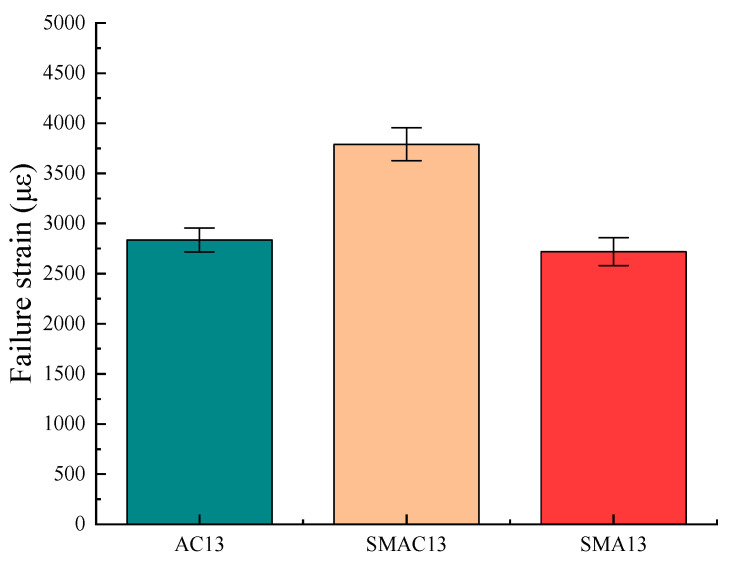
Results of beam bending test.

The three-point beam bending test at −10 °C was performed to evaluate the performance of resistance to cracking at low temperature according to China standards [29]. Four specimens for each kind of asphalt mix were tested (Figure 11). The failure strain can be calculated using Equation (4). The values of failure strain for three kinds of asphalt mixes at −10 °C are shown in Figure 12.

(4) $$\varepsilon_{B}=\frac{6hd}{L^{2}}$$

where $\varepsilon_{B}$ is failure strain, *h* is the height of the section at midspan, d is the displacement at midspan, and L is the span of the specimen.

From Figure 12, all asphalt mixes met the requirements for low-temperature performance in the specifications [21]. Also, the order of the low-temperature performance of the three kinds of asphalt mixes is SMAC13, AC13, and SMA13 in terms of failure strain values. The failure strain value for SMAC13 was 1.39 times that of SMA13 and 1.34 times that of AC13. The enhanced low-temperature cracking resistance of SMAC13 may be attributed to the utilization of limestone aggregate and fibers.

#### 4.2.3. Water Stability

**Figure 13:**
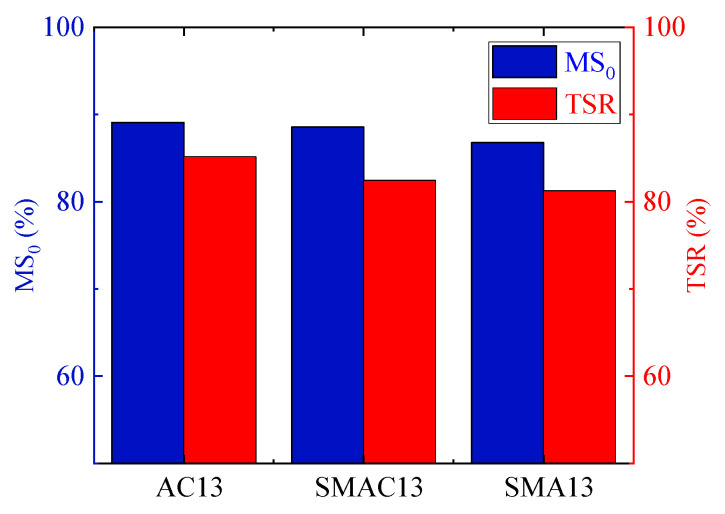
Comparison of water stability for three kinds of asphalt mixes.

The water stability of three kinds of asphalt mixes was evaluated using the Marshall immersion test and freeze–thaw splitting test. The tests were conducted as per China standards [29]. The indexes of two tests were residual stability (${MS}_{0}$) and the freeze–thaw splitting strength ratio (TSR), which were calculated using Equations (5) and (6). The results of the Marshall immersion test and freeze–thaw splitting test are shown in Figure 13.

(5) $$MS_{0}=\frac{MS_{1}}{MS}\times 100$$

where ${MS}_{1}$ is the average Marshall stability under wet immersion conditions (kN). MS is the average Marshall stability under standard conditions (kN).

(6) $$TSR=\frac{TS_{c}}{TS_{d}}\times 100$$

where ${TS}_{c}$ is the average indirect tensile strength of the conditioned group and ${TS}_{d}$ is the average indirect tensile strength of the dry group.

From Figure 13, it is evident that AC13 shows the highest ${MS}_{0}$ and TSR, followed by SMAC13 then SMA13, but the difference among them is small. The results indicate that the water stability of SMAC13 is between SMA13 and AC13. Also, all asphalt mixes meet the requirements for water stability in the specifications [21].

#### 4.2.4. Economic Analysis

Based on the results of the previous gradation design, a brief economic analysis for three kinds of asphalt mixes was performed. In this study, limestone aggregates (LAs) and 5.0% asphalt aggregate ratio were used in AC13, limestone aggregates, 5.5% asphalt aggregate ratio, and 0.2% fiber were used in SMAC13, and basalt aggregates (BAs), 6.2% asphalt aggregate ratio, and 0.3% fiber were used in SMA13. The costs of three kinds of asphalt mixes were estimated according to their respective proportions of mineral aggregate and the unit costs of raw materials, as shown in Table 11.

As presented in Table 11, the unit price ranking of three kinds of asphalt mixes is SMA13, SMAC13, and AC13. The cost of SMAC13 is 8.5% higher than that of AC13 and 25.4% lower than that of SMA13. Based on the comparison of pavement performance and costs of three kinds of asphalt mixes, it can be concluded that SMAC13 developed in this study has better cost-effectiveness and might be an alternative for asphalt pavement surface layers on high-grade highways and urban roads.

## 5. Conclusions

On the basis of design of asphalt mix proportion and laboratory tests of conventional performance, a new kind of hot mix asphalt named SMAC13 was developed. The following main conclusions can be drawn from this study.

1. The gradation range of SMAC13 was proposed through the design of the grading curve family for SMAC13 and verification of skeleton interlock of coarse aggregates of these gradation curves. On the whole, the middle section of the gradation range (2.36 mm–9.5 mm) is above the upper limit of gradation for SMA13, while the tail section (2.36 mm–0.075 mm) and head section (9.5 mm–16 mm) are within the gradation range of AC13.

2. Based on the experimental results of the wheel tracking test, beam bending test, Marshall immersion test, and freeze–thaw splitting test, the quality sequence of three kinds of asphalt mixes is SMA13, SMAC13, and AC13 in terms of high-temperature performance. The quality sequence of low-temperature performance is SMAC13, AC13, and SMA13, and the quality sequence of water stability is AC13, SMAC13, and SMA13.

3. Overall, SMAC13 shows the best low-temperature cracking resistance, and the high-temperature rutting resistance and moisture susceptibility are between SMA13 and AC13. Furthermore, the cost of SMAC13 is 25% less than that of SMA13. As such, SMAC13 can be used as an alternative for the surface course of asphalt pavement due to its higher performance and lower cost.

## Figures and Tables

**Table 1:** Basic properties of coarse aggregates.

Indexes	Basalt		Limestone		Specifications
	5–10 mm	10–15 mm	5–10 mm	10–15 mm	
Apparent specific gravity	2.894	2.892	2.719	2.683	>2.60
Bulk specific gravity	2.753	2.797	2.597	2.597	>2.50
Water absorption (%)	1.3	1.1	1.4	1.2	≤2.0
Crushed stone value (%)	11.2		19.9		≤26
L.A. abrasion (%)	8.7		17.1		≤28
Polished stone value (PSV)	51		44		≥40

**Table 2:** Basic properties of limestone and basalt fine aggregates.

Indexes	Basalt		Limestone	Specifications
	0–3 mm	3–5 mm	0–5 mm	
Apparent specific gravity	2.886	2.879	2.791	>2.50
Water absorption (%)	2.4	2.0	2.2	—

**Table 3:** Basic physical properties of mineral filler.

Item		Unit	Test Results	Specifications
Moisture content		%	0.3	≤1.0
Apparent relative density		—	2.821	≥2.50
Exterior appearance		—	No agglomeration	No agglomeration
Heat stability		—	Qualified	Qualified
Grain size range	<0.6 mm	%	100	100
	<0.15 mm	%	91.4	90–100
	<0.075 mm	%	79.6	75–100

**Table 4:** Basic properties of SBS-modified asphalt.

Indexes	Unit	Specifications	Test Results
Penetration (25 °C, 100 g, 5 s)	0.1 mm	40–60	58
Softening point	°C	≥55	80.2
Ductility (5 cm/min, 5 °C)	cm	≥30	30.7
Brookfield viscosity (135 °C)	Pa·s	<3	2.5
Storage stability (48 h softening point difference)	°C	≤2.5	2.0

**Table 5:** Basic properties of lignin fiber.

Item	Unit	Specifications	Test Results
Fiber length	mm	≤6	4.6
Ash content	%	18 ± 5	17.2
pH value	—	7.5 ± 1.0	7.3
Oil absorption	—	≥5 times weight of fiber	5.4 times
Moisture content	%	≤5	3.1

**Table 6:** Passing rate of BSS of family grading curves.

Gradations	1	2	3	4	5	6	7
Passing rate (%)	35.0	31.0	29.5	28.0	26.5	25.0	20.5

**Table 7:** Initial asphalt aggregate ratios of SMAC13.

Gradations	$\gamma_{\mathrm{sb}}$	$\gamma_{\mathrm{se}}$	*P*_a_ at Different *VMA* Values (%)		
			*VMA* = 14%	*VMA* = 14.5%	*VMA* = 15%
1	2.683	2.728	5.0	5.3	5.5
2	2.672	2.719	5.1	5.3	5.6
3	2.668	2.715	5.1	5.4	5.6
4	2.663	2.712	5.1	5.4	5.6
5	2.659	2.708	5.2	5.4	5.7
6	2.655	2.705	5.2	5.4	5.7
7	2.642	2.696	5.3	5.5	5.8

**Table 8:** Values of VCA and VCA_mix_.

Gradation No.	*VCA* (%)	$\gamma_{CA}$	$P_{CA}$ (%)	${VCA}_{mix}$ (%)
1	39.08	2.644	62.25	42.39
2	39.14	2.636	66.03	38.88
3	39.21	2.633	67.46	37.84
4	39.22	2.630	68.90	36.75
5	39.30	2.627	70.33	35.66
6	39.37	2.625	71.77	34.63
7	39.70	2.623	76.08	31.43

**Table 9:** Gradation range of SMAC13.

Sieve Size (mm)	Upper Limit of Gradation (%)	Lower Limit of Gradation (%)
16	100	100
13.2	100	90
9.5	83	73
4.75	47	35
2.36	32	22
1.18	26	17
0.6	21	12
0.3	16	9
0.15	13	7
0.075	10	6

**Table 10:** SMAC13 volumetric indicators and drain-down.

Fiber Content (%)	Asphalt Aggregate Ratio (%)	$\gamma_{t}$	$\gamma_{b}$	*VV* (%)	*VMA* (%)	*VFA* (%)	Drain-Down (%)
0.0	5.8	2.486	2.391	3.80	15.16	74.91	0.189
0.2	5.8	2.483	2.394	3.58	15.23	76.50	0.092

**Table 11:** Costs of three kinds of asphalt mixtures.

Materials	LA	BA	Asphalt	Fiber	AC13	SMAC13	SMA13
Unit price (yuan/ton)	85	170	4930	3000	315.71	342.62	459.28

## Data Availability

Data will be made available on request.
